# Evolutionary Origins and Functions of the Carotenoid Biosynthetic Pathway in Marine Diatoms

**DOI:** 10.1371/journal.pone.0002896

**Published:** 2008-08-06

**Authors:** Sacha Coesel, Miroslav Oborník, Joao Varela, Angela Falciatore, Chris Bowler

**Affiliations:** 1 Cell Signalling Laboratory, Stazione Zoologica ‘Anton Dohrn’, Villa Comunale, Naples, Italy; 2 Institute of Parasitology, Biology Centre of the Academy of Sciences of the Czech Republic and Faculty of Science, University of South Bohemia, Ceské Budejovice, Czech Republic; 3 Centre of Marine Sciences, University of Algarve, Faro, Portugal; 4 CNRS UMR8186, Department of Biology, Ecole Normale Supérieure, Paris, France; Yale University, United States of America

## Abstract

Carotenoids are produced by all photosynthetic organisms, where they play essential roles in light harvesting and photoprotection. The carotenoid biosynthetic pathway of diatoms is largely unstudied, but is of particular interest because these organisms have a very different evolutionary history with respect to the Plantae and are thought to be derived from an ancient secondary endosymbiosis between heterotrophic and autotrophic eukaryotes. Furthermore, diatoms have an additional xanthophyll-based cycle for dissipating excess light energy with respect to green algae and higher plants. To explore the origins and functions of the carotenoid pathway in diatoms we searched for genes encoding pathway components in the recently completed genome sequences of two marine diatoms. Consistent with the supplemental xanthophyll cycle in diatoms, we found more copies of the genes encoding violaxanthin de-epoxidase (VDE) and zeaxanthin epoxidase (ZEP) enzymes compared with other photosynthetic eukaryotes. However, the similarity of these enzymes with those of higher plants indicates that they had very probably diversified before the secondary endosymbiosis had occurred, implying that VDE and ZEP represent early eukaryotic innovations in the Plantae. Consequently, the diatom chromist lineage likely obtained all paralogues of *ZEP* and *VDE* genes during the process of secondary endosymbiosis by gene transfer from the nucleus of the algal endosymbiont to the host nucleus. Furthermore, the presence of a ZEP gene in *Tetrahymena thermophila* provides the first evidence for a secondary plastid gene encoded in a heterotrophic ciliate, providing support for the chromalveolate hypothesis. Protein domain structures and expression analyses in the pennate diatom *Phaeodactylum tricornutum* indicate diverse roles for the different ZEP and VDE isoforms and demonstrate that they are differentially regulated by light. These studies therefore reveal the ancient origins of several components of the carotenoid biosynthesis pathway in photosynthetic eukaryotes and provide information about how they have diversified and acquired new functions in the diatoms.

## Introduction

The brown unicellular diatoms (Bacillariophyceae) constitute a successful group in the phytoplankton community estimated to be responsible for 30 to 40 % of marine primary production [1]. Their role in global carbon cycling is predicted to be comparable to that of all terrestrial rain forests combined [2], [3], and they therefore represent an ecologically important group of bloom-forming phytoplankton. Diatoms can be classified into two major groups based on the symmetry of the frustule [4]. Centric diatoms are radially symmetrical and mostly planktonic, whereas pennate diatoms have a bilateral symmetry. Fossil records indicate that centric diatoms appeared at least 180 million years ago (Ma), whereas raphid pennate diatoms are thought to have evolved from centric diatoms prior to 90 Ma [5].

The importance of diatoms in marine ecosystems led to the sequencing of the nuclear, plastid and mitochondrial genomes of the centric diatom *Thalassiosira pseudonana*. The 34.5 Mb nuclear genome of this diatom was predicted to contain over 11,000 genes distributed on 24 chromosomes [6]. About half of the genes in this genome could not be assigned functions on the basis of similarity to genes in other organisms, and these genes may encode proteins involved in diatom-specific processes. The other half of the predicted genes have similar alignment scores to their closest homologs in either plant, red algal or animal genomes, which underscores the novel evolutionary history of diatoms (see below) [6]–[8]. More recently, the pennate diatom *Phaeodactylum tricornutum* was chosen as the second diatom for whole genome sequencing, partly because the physiology of this species has been studied for decades and because many molecular tools have been developed for this alga [9], [10]. Comparison between the two diatoms may also shed light on the evolution of diatoms. The nuclear *P. tricornutum* genome (27.4 Mb) is slightly smaller than the *T. pseudonana* genome but contains approximately the same number of genes and chromosomes (unpublished data). A recently assembled, and still expanding, diatom expressed sequence tag (EST) database [11], [12] has been linked to the *T. pseudonana* and *P. tricornutum* genome databases, which aids the prediction of genes and also provides information on gene expression profiles.

**Figure 1:**
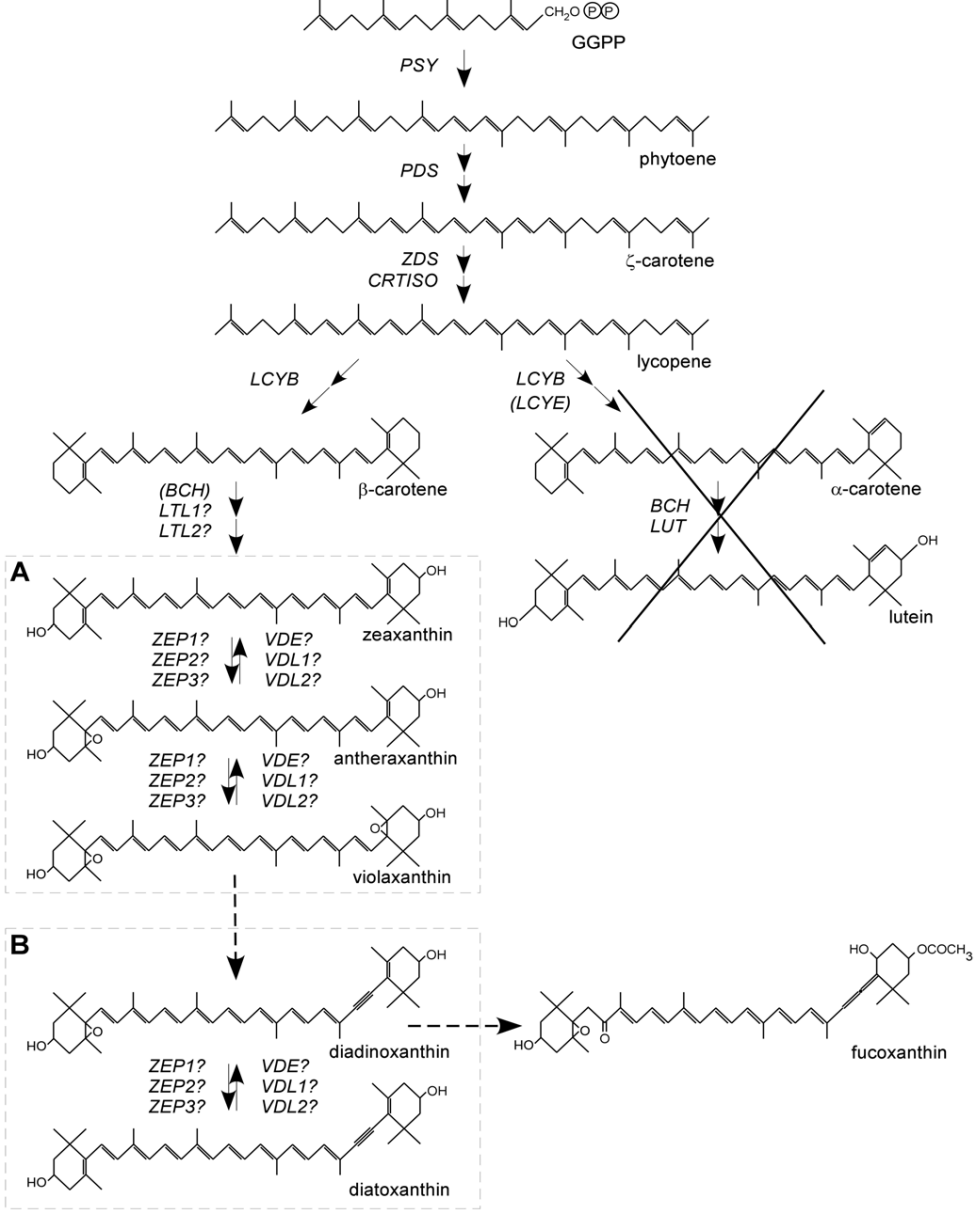
Hypothesized carotenoid biosynthetic pathway in diatoms. The genes identified in this study, phytoene synthase (*PSY*), phytoene desaturase (*PDS*), ξ-carotene desaturase (*ZDS*), lycopene β-cyclase *(LCYB*), β-carotene hydroxylase (*BCH*), lutein deficient-like (*LTL*), zeaxanthin epoxidase (*ZEP*), violaxanthin de-epoxidase (*VDE*) and violaxanthin de-epoxidase-like (*VDL*), are indicated. The BCH-encoding gene is absent in the *P. tricornutum* genome, *ZEP3* and *VDL2* are absent in the *T. pseudonana* genome. The two xanthophyll cycles are boxed, A) the violaxanthin cycle and B) the diadinoxanthin cycle. α-carotene and lutein are not produced by diatoms. Dashed arrows indicate hypothetical conversion steps, according to Lohr and Wilhelm (1999, 2001).

Diatoms are believed to have obtained their plastid from a secondary endosymbiosis between a heterotrophic eukaryote and an ancient red alga. This event is postulated to have occurred at least 800 Ma [13]–[15] and may subsequently have given rise to the chromalveolates, which includes the group of algae collectively called Chromista. The chromist algae, Haptophyta (e.g., coccolithophorids), Cryptophyta (e.g., *Guillardia theta*), Heterokonts (diatoms and brown algae), and tertiary red-symbiotic dinoflagellates, all use chlorophyll *a*, chlorophyll *c,* and fucoxanthin as light-harvesting pigments [16]. Fucoxanthin, a carotenoid presumably derived from β-carotene (Fig. 1), absorbs light in the blue range of the light spectrum and is largely responsible for the characteristic brown color of chromist algae. The success of chromist algae may be explained by their ability to maintain photosynthetic activity in the blue-light-dominated oceanic environment [17]–[19]. In particular, diatoms have a huge capacity to dissipate excess absorbed light energy and their non-photochemical quenching (NPQ) levels can be as much as five times the levels registered for higher plants [20]. Moreover, diatoms are able to apply this photoprotective mechanism without significantly altering their light harvesting capacity [21], which allows them to maintain high growth rates over a wide range of light intensities [22].

The working model for energy-dependent NPQ (qE) in higher plants is based on the action of two oxygenated carotenoids, zeaxanthin and violaxanthin (Fig. 1). Essentially, the xanthophyll zeaxanthin binds protonated light-harvesting complex (LHC) proteins which may subsequently lead to a conformational change of the LHC and thereby allow the excess energy to be dissipated as heat [23]–[25]. The xanthophyll cycle allows plants to rapidly and tightly control their zeaxanthin content depending on the actual light conditions [26], [27]. The cycle is performed by two enzymes: zeaxanthin epoxidase (ZEP) and violaxanthin de-epoxidase (VDE) [28], [29]. Under dark/low light conditions, ZEP converts zeaxanthin, via antheraxanthin, to violaxanthin in two subsequent epoxidation reactions and when the photosynthetic apparatus receives too much light, VDE converts violaxanthin back to zeaxanthin in a reverse reaction (Fig. 1). In diatoms, the role of zeaxanthin has been supplemented by another xanthophyll molecule called diatoxanthin [Dtx; 30,31], and they appear to lack the PsbS protein, which has been implicated in the mechanism of NPQ in higher plants [23]–[25]. The diadinoxanthin cycle (Fig. 1) comprises the reversible conversion of diadinoxanthin (Ddx) and diatoxanthin (Dtx) [32], [33]. Curiously, the ancestors of primary plastids (cyanobacteria) and secondary brown plastids, which are thought to originate from an engulfed rhodophyte, do not perform xanthophyll cycling, so the evolution of violaxanthin de-epoxidase and zeaxanthin epoxidase in the green and brown photosynthetic lineages is of particular interest.

In the current study the fully sequenced genomes of *T. pseudonana* and *P. tricornutum* were used to identify genes putatively involved in carotenoid biosynthesis and special attention was given to genes of the two diatom xanthophyll cycles. Candidate genes were subsequently compared with known sequences from other organisms in order to study the evolutionary history of carotenoid biosynthesis from the ancestral cyanobacterium to the modern diatoms, and also from centric diatoms to raphid pennate diatoms. A number of environmental conditions, which may control the expression of diatom carotenoid biosynthesis-related genes, were assessed experimentally and *in silico* by means of quantitative real-time RT-PCR and by analysis of the *Phaeodactylum* Digital Gene Expression Database (http://www.biologie.ens.fr/diatomics/EST3).

## Results and Discussion

The *P. tricornutum* and *T. pseudonana* nuclear genomes were examined for putative genes of the carotenoid biosynthetic pathway. The diatom EST database (http://www.biologie.ens.fr/diatomics/EST3), containing over 130,000 ESTs from *P. tricornutum*, was of great importance for accurate gene annotation. All selected gene models contain start and stop codons, unless stated otherwise, as well as putative upstream targeting sequences as determined by SignalP v3.0 and ChloroP v1.1 [34]. A summary of diatom genes putatively encoding carotenoid pathway components is shown in Table 1, and details concerning the early carotenoid biosynthetic pathway are given in the Supplementary Information. In short, our findings indicate that diatoms most likely have inherited the genes involved in the early reactions of carotenoid biosynthesis (up to β-carotene) from the algal endosymbiont. However, the gene encoding β-carotene hydroxylase (BCH), responsible for the hydroxylation of β-carotene into zeaxanthin (Fig. 1), is absent in the *P. tricornutum* genome and only a partial sequence is present in the *T. pseudonana* genome. This enzymatic reaction may therefore be catalyzed by other unrelated enzymes in diatoms, such as LUT-like P450 proteins (see Supplementary Information S1 and Figure S1). Due to their special interest in diatoms the enzymes involved in the xanthophyll cycle were studied in more detail.

**Table 1:** Genes of the *P. tricornutum* and *T. pseudonana* carotenoid biosynthetic pathway.

protein	ID Phatr2	length (aa)	introns	SignalP (ChloroP***)	ID Thaps3	id/sim (%)	best hit NCBI	Accession	id/sim (%)
PSY1	56481	505	1	0.881 (0.526)	268908	61/71	*Pavlova lutheri*	ABA55571	45/61
PSY2	np	-	-	-	258309*	-	*Zea mays*	AAR31885	32/45
PDS1	45735	624	0	0.985 (0.531)	6524	81/89	*Ostreococcus lucimarinus*	ABO98307	74/83
PDS2	55102	589	1	0.999 (0.479)	1383	52/68	*Gentiana lutea*	BAB8246	57/74
ZDS	53974	591	0	1.000 (0.564)	37288	83/91	*Lyngbya sp. PCC 8106*	EAW35008	59/73
CRTISO	*up to 6 related genes+1 gene with closer homology to crtI*								
LCYB	56484	659	1	0.994 (0.561)	261407	57/70	*Capsicum annuum*	CAA60119	32/48
BCH	np	-	-	-	263437*	-	*Glycine max*	AAS88426	39/54
LUT-like1	50101	644	1	0.987 (0.564)	9541	75/85	*Ostreococcus tauri*	CAL50459	50/66
LUT-like2	26422	769	0	0.560 (0.474)	36235	69/81	*Skeletonema costatum*	AAL73435	70/82
ZEP1	45845	565	0	0.997 (0.574)	269147	66/75	*Chlamydomonas sp.* W80	AAO48941	33/47
ZEP2	56488	604	1	1.000 (0.566)	261390	68/75	*Vigna unguiculata*	BAB11934	31/45
ZEP3	56492	557	2	0.988 (0.560)	np	-	*Arabidopsis thaliana*	AAG38877	34/49
VDE	44635	437	0	0.902 (0.518)	7677	59/73	*Chrysanthemum × morifolium*	BAE79554	42/61
VDL1	46155	453	0	0.999 (0.464)	22076	74/82	*Spinacia oleracea*	CAB59211	20/34
VDL2	45846	555	1	0.997 (0.531)	np	-	*Medicago truncatula*	ABO83847	17/25
VDR	56450	587	0	0.912 (0.512)	270211	65/80**	*Oryza sativa*	EAZ13333	42/58**

The Phatr2 genome browser was used to identify *P. tricornutum* gene models encoding putative carotenoid biosynthetic enzymes (see text for abbreviations), and the following parameters are given: the protein identification number (ID) of the best gene model, the length of the immature (including putative signal peptide) protein, the number of introns, the probability of the presence of a signal peptide as determined by SignalP v3.0 and, in parentheses, the chloroplast targeting prediction score as determined with ChloroP v1.1. The Thaps3 genome browser was used to identify the *T. pseudonana* homologs and the protein IDs are shown together with the identity/similarity to the respective *P. tricornutum* gene. The identity/similarity was determined after aligning the mature protein sequences with ClustalW using the BLOSUM62 similarity matrix. BLASTP searches on the NCBI sequence browser were performed on 24-08-2007 and the NCBI accession number and identity/similarity with the respective *P. tricornutum* genes are given. When the Phatr2 gene model is absent, the Thaps3 model was used instead. When a gene model was not full length, the alignment was trimmed to the shortest sequence.

np) not present, ^*^) gene model not complete, ^**^) cleavage site of VDE was used, ^***^) Cut-off value of 0.500 used for ChloroP1.1.

### Domain structures of diatom violaxanthin de-epoxidases

Violaxanthin de-epoxidase (VDE)-encoding genes have been identified in a broad range of plants and they form a highly conserved family. VDE is characterized as a soluble protein located in the thylakoid lumen and the optimal VDE activity is found between pH 5.0 and 5.2 *in vivo* [26], [35]. We have previously reported the presence of two VDE-encoding genes in the centric diatom *T. pseudonana* [8], one of which is similar to the *VDE* of higher plants, while the other (designated as violaxanthin de-epoxidase-like; *TpVDL*) is more distantly related. We had proposed that the first might be involved in the conventional xanthophyll cycle found also in green photosynthetic eukaryotes, while the latter may be more specialized in the chromist-specific diadinoxanthin cycle. Analysis performed in this work indicate that the *P. tricornutum* genome contains one *VDE* gene and two *VDE-like* genes, designated as *PtVDE* and *PtVDL1* and *2*, respectively (Table 1).

**Figure 2:**
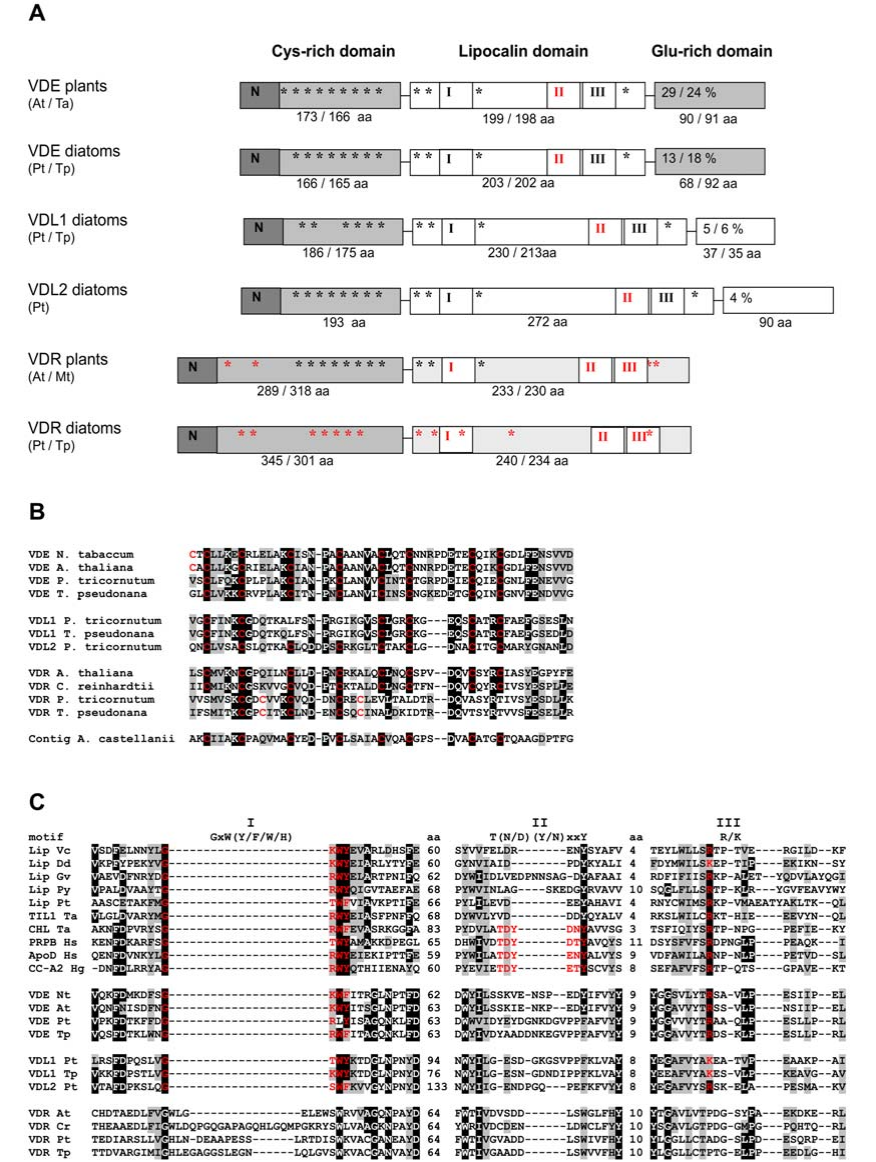
Domain structure of violaxanthin de-epoxidases and related proteins. A) Schematic representation of VDE, VDL and VDR proteins (not to scale). Three different domains are shown; the cysteine-rich domains include the N-terminal targeting sequence. Black and red asterisks indicate the positions of conserved and alternative cysteine residues, respectively. The central lipocalin domain contains the lipocalin binding fold. Conserved and divergent lipocalin motifs (roman numbers) are given in black and red, respectively. The size of the lipocalin motif was determined by sequence alignment of VDE sequences and a representative group of lipocalin proteins. The C-terminal glutamic acid-rich domain indicates the percentage of Glu residues in this domain. B) Alignment of the N-terminal cysteine-rich domains of several plant and diatom VDEs. Also included is a sequence derived from the amoeba *Acanthamoeba castellanii.* C) Alignment of the lipocalin motifs I, II and III of several different lipocalin VDE, VDL and VDR proteins. The distance (in amino acids) between the three lipocalin motifs is also indicated. The lipocalin motif consensus sequences, as derived from kernel lipocalins (Flower, 1996), are indicated above the alignment and conserved motifs within the alignment are indicated in red. The abbreviations used are: Lip, lipocalin; TIL, temperature induced lipocalin; CHL, chloroplastic lipocalin; PRBR, plasma retinol-binding protein precursor; CC, crustacyanin; At, *Arabidopsis thaliana;* Cr, *Chlamydomonas reinhardtii;* Dd, *Dictyostelium discoideum;* Gv, *Gloeobacter violaceus;* Hg, *Homarus gammarus; Hs, Homo sapiens;* Mt, *Medicago truncatula;* Nt, *Nicotiana tabacum;* Pt, *Phaeodactylum tricornutum;* Py, *Porphyra yezoensis;* Ta, *Triticum aestivum;* Tp, *Thalassiosira pseudonana;* Vc, *Vibrio cholerae.*

A comparison between the domain structures of the plant and diatom VDE proteins shows that the diatom proteins are relatively similar to the plant counterparts and consist of a cysteine-rich N-terminal domain, a lipocalin domain, and a C-terminal glutamic acid-rich domain (Fig. 2). The cysteine residues in the first domain can form one or more disulfide bridges [28] and this domain seems essential for VDE function because deletion of any region in this domain leads to a total loss of VDE activity [36], [37]. The second domain, which is thought to bind the xanthophyll molecule in the all-*trans* configuration, shows similarity to the eight-stranded β-barrel structure of the lipocalin protein family [38]. The small proteins (±200 aa) of this family are highly divergent in amino acid composition but are conserved in their tertiary structure, which allows them to bind small hydrophobic molecules [39]. Both these domains are generally well conserved between diatoms and plants, indicating that these proteins show similar folding and can bind the same molecules. However, the C-terminal Glu-rich domain is considerably less conserved between diatoms and plants: whereas the C-terminal domain of plant VDEs contains an average of 47% charged residues of which about 25% are glutamic acid residues, the percentage of charged amino acids in the PtVDE and TpVDE domains is 29 and 37% (13 and 18% Glu), respectively. Partial protonation of the glutamic acid-rich domain is thought to increase the binding of VDE to the thylakoid membrane [28], [40], and the divergence of this C-terminal domain may likely affect the pH-dependent binding of the diatom VDE to the thylakoid membrane [41], [42].

In the diatom VDL1 and VDL2 sequences, the Glu-rich domain has been completely replaced by an uncharged C-terminal domain. Therefore it is possible that the activation and/or localization of VDL proteins may be different than the VDE proteins. The lipocalin domain of the VDL proteins is considerably larger (Fig. 2), but they nevertheless contain the two typical lipocalin motifs (I and III), which are important for the correct folding of the β-barrel structure. Extensive searches in the available online genome and EST databases showed that genes containing this larger lipocalin domain are found only in chlorophyll *c-*containing chromist algae (see below). We therefore consider it possible that the VDL enzymes may be able to more efficiently bind, and eventually de-epoxidize, brown algal-specific molecules such as diadinoxanthin. However, it has to be noted that an *in vitro* enzyme assay with spinach VDE demonstrated that the conventional VDE enzyme is also able to make the conversion of diadinoxanthin, albeit with a 30% reduced efficiency as compared to violaxanthin [43], so without experimental evidence we cannot rule out the possibility that the VDL proteins may have other unrelated or additional functions.

The genome of *Chlamydomonas reinhardtii* lacks a copy of the *VDE* gene (possibly due to incomplete sequencing), although careful scanning of the *C. reinhardtii* genome for VDE led to the discovery of a novel gene designated *VDE-related* (*VDR*). VDR does not contain the typical VDE protein family signature (pfam 07137) which spans the lipocalin domain (Fig. 2) and does not contain a C-terminal domain, but nevertheless it still shares sequence similarity with VDE (*A. thaliana* VDE and VDR: 12% id, 27% sim). Following this first identification, *VDR* homologues were subsequently found in higher plants as well, and it appears that this gene of unknown function is ubiquitously present in all green photosynthetic eukaryotes. We found that the two diatom genomes also contain a copy of the *VDR* gene, and that the encoded amino acid sequences are relatively similar to the plant counterpart (Table 1), except for the position of the conserved cysteine residues (Fig. 2), which probably has implications for the folding of the protein.

Even though VDE, together with ZEP, were the first lipocalin-like proteins to be identified in plants [38], [44], there is still considerable debate as to whether VDE can be truly ascribed to the lipocalin protein family because of the great divergence in size, function, and genomic organization between lipocalins and VDEs [45]-[47]. The most accepted evolutionary scenario for the appearance of plant VDEs involves a gene fusion event between a lipocalin and another gene, together giving rise to the larger VDE protein with a newly acquired function [45], [46]. To find the ancestral lipocalin sequence, which may have given rise to the modern VDEs, Charron *et al*. [46] searched 14 complete and 2 partial cyanobacterial genomes, and found only 1 cyanobacterial species that contained a lipocalin gene. This particular species, *Gloeobacter violaceous* PCC7421, belongs to the most ancient members of the cyanobacterial lineage, which do not possess thylakoid membranes but instead use the plasma membrane to attach the phycobilisomes [48]. Charron *et al*. [46] speculated that the *G. violaceous* lipocalin might be the ancestor of recently identified chloroplastic lipocalins (CHL), VDEs and ZEPs of higher plants. However, there is currently no solid phylogenetic evidence to support the role of the cyanobacterial lipocalin in the occurrence of the xanthophyll cycle genes.

In order to find evidence for the proposed gene-fusion event between a lipocalin and a second gene, we used the well-conserved N-terminal cysteine-rich domain of VDE to search the microbial and protozoan genomes available at NCBI using BLAST. While the 915 microbial genomes did not yield any positive hits, a relatively conserved Cys-rich domain (>40% similarity) was present in the protozoan genomes of *Phytophthora sojae* and *P. ramorum*, *Entamoeba histolytica*, *Toxoplasma gondii*, and *Trypanosoma cruzi*. A similar search against the Taxonomically Broad EST Database (TBestDB) was performed, and an especially well conserved Cys-rich domain in an EST contig encoding a multi-domain protein of the protozoa *Acanthamoeba castellanii* was found. To illustrate the homology, this sequence was placed within the Cys-rich domain alignment of Figure 2B. Because protists, but not cyanobacteria, contain well-conserved expressed Cys-rich-encoding proteins in their genomes, the N-terminal domain of the VDE-encoding gene may have originated from the protozoan host. Furthermore, because only chlorophyll *b* and *c*-containing photosynthetic eukaryotes possess this Cys-rich domain in conjunction with a lipocalin domain, it can be hypothesized that VDE is the result of a gene-fusion event which probably occurred relatively early after the primary endosymbiosis of the cyanobacterium and the host cell.

### Phylogeny of violaxanthin de-epoxidases

The identification of plant-like VDEs in diatoms and other unicellular brown algae is most easily explained by the presence of VDE in the common ancestor of the green and red lineages. However, the main light-harvesting complexes of rhodophytes, the phycobilisomes, do not use a zeaxanthin-dependent NPQ mechanism to dissipate excess-absorbed light [49], [50], and physiological experiments on a wide range of different red algal species indicate that red algae do not convert violaxanthin to zeaxanthin upon illumination [50], although some red algal species are able to produce antheraxanthin and violaxanthin [50], [51]. Furthermore, no *VDE* sequence evidence from a red alga has been forthcoming from the *C. merolae* genome, nor from the EST sequences of the red macroalga *Porphyra yezoensis* (data not shown).

**Figure 3:**
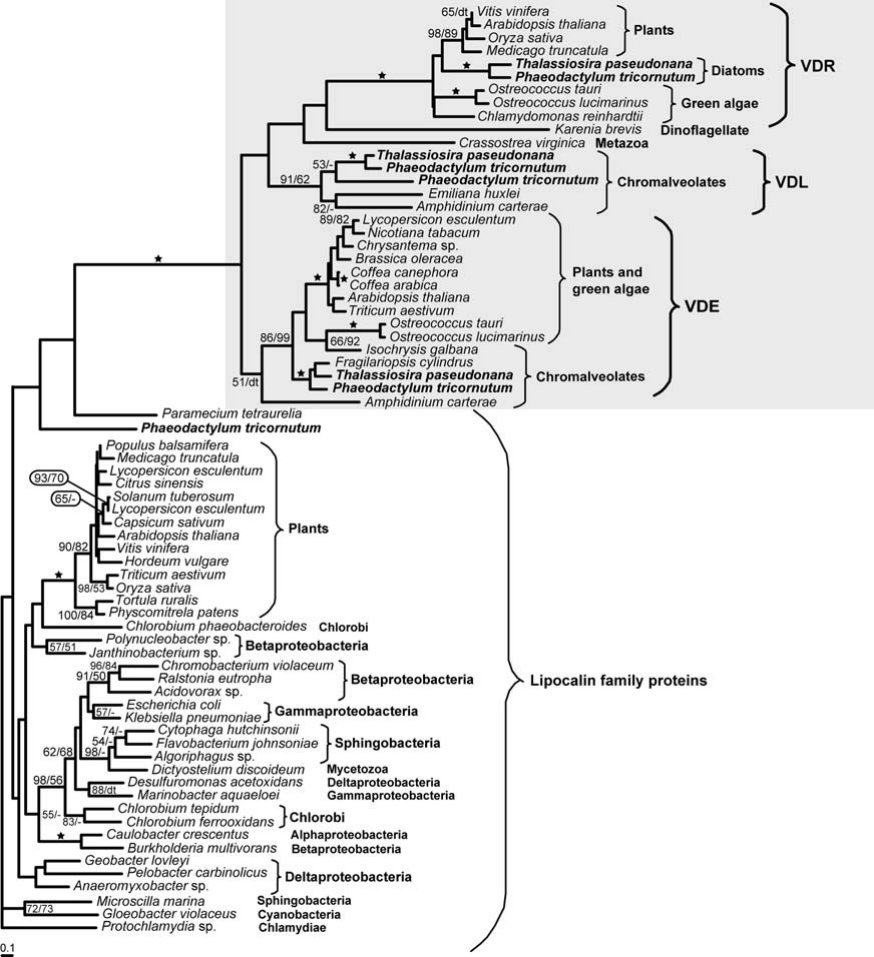
Maximum likelihood phylogenetic tree of violaxanthin de-epoxidases and related proteins. A maximum likelihood phylogenetic tree (loglk = −13981.66253) as inferred from amino acid sequences (141 amino acid characters) of violaxanthin de-epoxidases and related proteins was computed using WAG model for amino acid substitution (selected by PROTTEST) with discrete gamma distribution in four categories. All parameters (gamma shape = 2.158; proportion of invariants = 0.000) were estimated from the dataset. Numbers above branches indicate ML/NJ bootstrap supports. ML bootstraps were computed using the above mentioned model in 300 replicates. An NJ tree was inferred using AsaturA program with cutoff value 0.906 and 1000 replicates. Black stars indicate both bootstraps over 90%. Nodes that display different NJ topology than the one obtained by ML are indicated by “dt”.

A phylogenetic tree of the diatom and plant VDEs and related genes was generated and rooted by related lipocalin family proteins from both prokaryotes and eukaryotes. As can be seen in the tree (Fig. 3), there is a considerable difference between the ancient lipocalins and VDEs, such that the ancestor of VDE proteins within these lipocalin proteins cannot be specified. The VDE proteins constituted three distinct clusters: VDE, VDL (VDE-like) and VDR (VDE-related) proteins. Two of these clusters (VDE and VDR) are composed of plant and diatom sequences with diatom sequences appearing on the root of each cluster (Fig. 3). Plant sequences are absent from the VDL cluster, which contains, in addition to diatom proteins, sequences from other chromalveolates, such as heterokonts and dinoflagellates (Fig. 3). A single metazoan VDL sequence from the eastern oyster *Crassostrea virginica,* which has been obtained from ESTs at NCBI, appeared within the VDL proteins; however, the position of this oyster sequence is not supported by bootstraps. Its position is also questioned by the fact that the NJ tree showed a different topology than the ML tree, placing *C. virginica* at the root of all VDE and related proteins (data not shown). The sequence from *C. virginica* is the only VDE sequence known from non-photosynthetic eukaryotes and, moreover, when Blast searched in NCBI it yields hits only from photosynthetic eukaryotes. We therefore have serious reservations about the identity of this sequence and speculate that it may be derived from contamination of *C. virginica* cDNA with material from eukaryotic algae.

Within the VDE+VDL+VDR cluster, VDE sequences seem to constitute an ancestral form, which is basal to the advanced sisters VDL and VDR (Fig. 3). It is obvious that VDL and VDR proteins have arisen by two duplication events. The second duplication could be associated with the secondary endosymbiosis, since it has happened only in chromalveolates. Alternatively, this particular paralogue was previously present only in red algae or has been lost from green plants.

Based on the tree topology we suggest that *VDE*, *VDL* and *VDR* genes are out-paralogues of the original *VDE* gene, with the duplication event preceding the speciation as well as the secondary endosymbiosis that led to the appearance of complex plastids. The fact that no homolog of VDE proteins has been found in prokaryotes suggests that they represent an ancient eukaryotic innovation. This is also supported by the fact that in cyanobacteria, the putative prokaryotic donor of genes encoding proteins of plastid functions, no VDE proteins have ever been identified and the lipocalin homolog is found only in a single cyanobacterial species *Gloeobacter violaceus*. We propose that VDE proteins have arisen in diatoms by the endosymbiotic gene transfer from the nucleus of the algal endosymbiont. In such a scenario, VDE proteins have been lost from the current rhodophytes. Plants lack VDL proteins, possibly because they lost it during evolution, similarly to rhodophytes, or because VDLs are for some reason only found in photoautotrophs derived from the secondary endosymbiosis. Based on the phylogenetic position of plant, diatom and other chromalveolate VDEs, we suggest that VDE proteins of green and brown algae share a common origin. Alternatively, the *VDE* gene may have been introduced in diatoms by a lateral gene transfer from a green alga. However, this scenario seems less likely to explain the widespread appearance of VDE throughout the brown clade, as seen by the presence of a VDE sequence in haptophytes.

### Domain structures of diatom zeaxanthin epoxidases

Zeaxanthin epoxidase is localized in the stromal side of the thylakoids, where it catalyzes the conversion of zeaxanthin to antheraxanthin and violaxanthin [52]. The gene encoding ZEP was first identified in a transposon-tagged *Nicotiana plumbaginifolia* abscisic acid (ABA)-deficient mutant, called *aba2* [29], and was found to encode a chloroplast-imported flavin-containing monooxygenase (FMO), containing an ADP-binding fold and an FAD-binding domain similar to prokaryotic aromatic-substrate monooxygenases. As predicted by Bugos *et al*. [38], VDE and ZEP share a similar basic tertiary structure based on a lipocalin domain.

**Figure 4:**
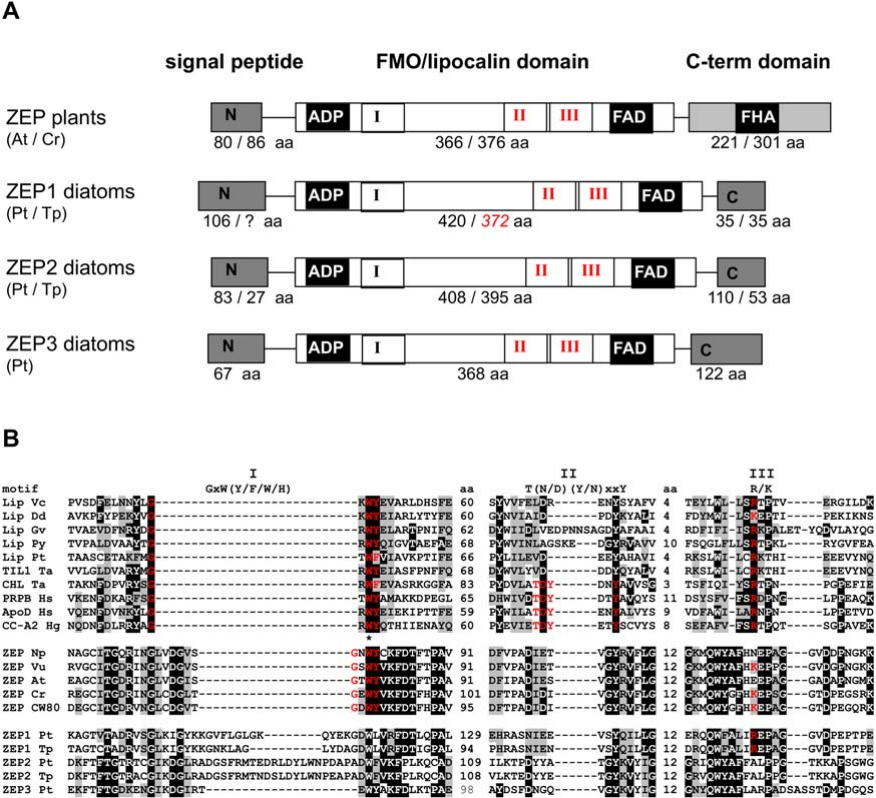
Domain structure of zeaxanthin epoxidases. A) Schematic representation of ZEP proteins (not to scale). Three different domains are discriminated; the N-terminal targeting sequence (note that the *TpZEP1* gene model is not complete), the central flavin-containing monooxygenase (FMO) domain containing ADP and FAD binding sites, and the C-terminal domain containing the FHA motif. Conserved and divergent lipocalin motifs (roman numbers) are given in black and red, respectively. The size of the lipocalin motif was determined by sequence alignment of ZEPs and a representative group of FMO proteins. B) Alignment of the lipocalin motifs I, II and III of several different lipocalins and ZEP protein sequences. The lipocalin motif consensus sequence, as derived from kernel lipocalins, is indicated above the alignment and conserved motifs within the alignment are indicated in red. The asterix indicates the Gly304 identified by Baroli *et al*. (2003). The distance (in amino acids) between the lipocalin motifs are also indicated. The abbreviations used are: Lip, lipocalin; TIL, temperature induced lipocalin; CHL, chloroplastic lipocalin; PRBR, plasma retinol-binding protein precursor; CC, crustacyanin; At, *Arabidopsis thaliana;* Cr, *Chlamydomonas reinhardtii; CW80, Chlamydomonas sp. W80;* Dd, *Dictyostelium discoideum;* Gv, *Gloeobacter violaceus;* Hg, *Homarus gammarus;* Hs, *Homo sapiens;* Np, *Nicotiana plumbaginifolia;* Pt, *Phaeodactylum tricornutum;* Py, *Porphyra yezoensis;* Ta, *Triticum aestivum;* Tp, *Thalassiosira pseudonana;* Vc, *Vibrio cholerae;* Vu, *Vigna unguiculata*.

We have previously reported the presence of two ZEP encoding genes in the centric diatom *T. pseudonana* [8]. As with VDE, this is one copy more than is generally found in higher plants and may again be a reflection of the two separate xanthophyll cycles found in heterokonts. As was the case for VDE, we found that the *P. tricornutum* genome contained a third copy of ZEP that is absent in the *T. pseudonana* genome (Table 1). As compared to the plant ZEPs, the amino acid region covering lipocalin motif I is considerably larger in the diatom ZEP1 and 2 proteins, but not in PtZEP3 (Fig. 4) and the motif I consensus sequence is not conserved in any of the diatom ZEPs. All diatom ZEPs lack the C-terminal forkhead-associated (FHA) domain that is normally found in plant ZEPs. In the diatom ZEP1 proteins this domain has been replaced with a conserved 35 amino acid sequence, whereas considerably larger sequences are present in the Pt and TpZEP2 and PtZEP3 proteins. Interestingly, a transmembrane region is predicted in the C-terminal domain of PtZEP3, which may have an effect on the localization and/or regulation of the diatom ZEP proteins. In this context it is interesting to note that the epoxidation kinetics of diatoxanthin in diatoms under low light conditions is generally faster than the epoxidation of zeaxanthin in higher plants and green algae [53], but the presence of a proton gradient almost completely inhibits this reaction in diatoms [53], [54]. This is in stark contrast with the epoxidation reaction of higher plants, which occurs in the dark as well as in the light, and the extent of zeaxanthin accumulation in plants depends largely on the activity of VDE. The mechanism behind the peculiar light-dependent ZEP activation/deactivation in diatoms is not yet understood, but it is possible that the divergent C-terminal domains of ZEP1, ZEP2 or ZEP3 may play a role.

Whereas zeaxanthin epoxidation in higher plants is not only important for xanthophyll cycling, but also for ABA synthesis, diatoms do not appear to synthesize this phytohormone. Diatoms however are thought to produce their major light harvesting carotenoid, fucoxanthin, from violaxanthin [Fig. 1; 55], [56], so the epoxidation reaction of zeaxanthin to synthesize fucoxanthin may require a different regulation than xanthophyll cycle-related epoxidation. The presence of two ZEP-encoding genes in *T. pseudonana* could also be a reflection of these two different processes, as was suggested by Wilhelm *et al.* [57]. The finding that *P. tricornutum* possesses a third copy of both VDE and ZEP is also very striking. Results presented by Goss *et al*. [53] indicated that the NPQ capacity of *P. tricornutum* is higher than *T. pseudonana,* whereas the diatoxanthin epoxidation rate under low light is considerably slower in *P. tricornutum*.

As seen in Figure 4, zeaxanthin epoxidases are FAD-dependent mono-oxygenases (FMO) containing a putative lipocalin fold. Even though it is possible to recognize at least two of the three lipocalin motifs within the FMO domain, the overall similarity between lipocalin proteins and the putative lipocalin fold of ZEP is extremely weak (<10% similarity). Therefore, a possible gene fusion event between a lipocalin and another gene, as was proposed above for the *VDE* genes, seems less likely to explain the evolution of *ZEP* genes. More probably, the lipocalin fold of ZEPs may have evolved in a process driven by steric constraints for binding the hydrophobic xanthophyll molecule. Since the FMO domain of ZEP proteins aligns very well with the amino acid sequence of other flavin-containing mono-oxygenases, except for the regions containing the lipocalin motifs, we propose that ZEP proteins may have evolved from an ancient flavin-containing monooxygenase-encoding gene.

### Phylogeny of zeaxanthin epoxidases

**Figure 5:**
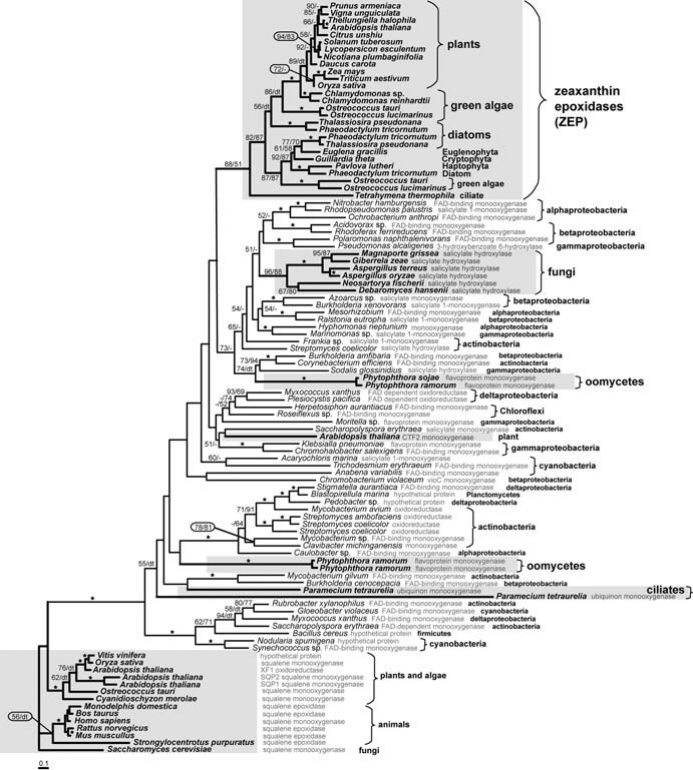
Maximum likelihood phylogenetic tree of zeaxanthin epoxidases and related proteins. A maximum likelihood phylogenetic tree (loglk = −48991.81356) as inferred from zeaxanthin epoxidases and related protein amino acid sequences (312 amino acid positions). The tree was computed using WAG model for amino acid substitution (selected by PROTTEST) with discrete gamma distribution in four categories. All parameters (gamma shape = 1.760; proportion of invariants = 0.010) were estimated from the dataset. Numbers above branches indicate ML/NJ bootstrap supports. NJ tree was inferred using AsaturA program with cutoff value 0.908 and 1000 replicates. Black stars indicate both bootstraps over 90%. The original annotation of ZEP related proteins is indicated. Nodes that display different NJ topology than the one obtained by ML, are indicated by “dt”.

To further explore the phylogenetic relationships of plant and diatom ZEPs, ML and NJ trees of ZEP proteins rooted with eukaryotic and prokaryotic squalene monooxygenases and epoxidases were generated (Fig. 5). It is evident that two copies of genes encoding ZEP proteins are generally encoded in photosynthetic eukaryotes, which represent out-paralogues originating in an ancient single gene duplication event. Both ZEP paralogues are still present in *Ostreococcus* spp. and in both diatoms, whereas higher plants retain only one copy of the gene, as do other chromalveolates such as *Guillardia theta* and *Pavlova lutheri*. *P. tricornutum* contains three possible out-paralogues of the *ZEP* gene (Fig. 5). It has to be noted that within the chromalveolate clade we can also find a ZEP sequence from *Euglena gracilis*, an organism belonging to the excavates and possessing a secondary green plastid. This may suggest that the distribution of *ZEP* genes is a result of early duplication of *ZEP* in a primary host, before the secondary endosymbiosis occurred. In this case, *ZEP* genes would have been duplicated in an ancestor of plants and algae containing a primary plastid, and these duplicates would have spread to photoautotrophs with secondary plastids by endosymbiotic gene transfer. In some lineages, various ZEP copies have been lost. This scenario is also supported by the presence of a single ZEP homologue in the ciliate *Tetrahymena thermophila,* which forms a monophyletic group at high confidence with other ZEP genes. Since *ZEP* genes have not been found in heterotrophs, its presence in this ciliate may serve as evidence for a plastid-containing ancestor of ciliates, thus supporting the chromalveolate hypothesis.

### Expression profiling of carotenoid biosynthesis-related genes in *P. tricornutum*

The *Phaeodactylum* Digital Gene Expression Database (v3.0) contains over 130,000 ESTs from *P. tricornutum* cells grown in 16 different conditions (http://www.biologie.ens.fr/diatomics/EST3). We have mined this extensive database for EST-derived contigs encoding genes of the carotenoid biosynthetic pathway, and have determined the frequency and distribution of each contig throughout the different EST libraries (Table 2). Although this type of expression analysis is not quantitative, it nevertheless provides useful information on general gene expression trends. The genes most represented in the EST database, *ZEP2*, *ZEP3* and *VDL1*, are all putatively involved in xanthophyll biosynthesis. Of these genes, *VDL1* appears to be the most constitutively transcribed.

**Table 2:** EST distributions of carotenogenesis-related genes in the *Phaeodactylum* Digital Gene Expression Database.

gene	contig	OS	OM	TM	TA	SM	SP	NR	NS	AA	UA	FS	BL	LD	HD	C1	C4	total (‰)
*PSY*	G41878	-	-	-	-	-	-	-	-	-	-	0.73	0.08	0.11	-	0.10	0.10	1.11
	G34382																	
*PDS1*	G35509	0.58	0.22	-	0.21	-	-	-	-	0.11	0.58	-	0.08	-	-	-	-	1.78
*PDS2*	G55102	-	0.22	-	-	-	0.13	-	0.11	-	-	-	0.25	-	0.28	-	-	0.99
*ZDS*	G9040	0.08	-	0.32	0.21	-	0.13	-	-	0.11	0.23	0.24	0.50	-	-	-	0.10	1.92
*LCYB*	G8835	-	0.22	-	0.21	-	0.13	0.28	-	0.11	-	-	-	0.22	-	-	0.19	1.36
	C193																	
*LTL1*	G16586	0.08	-	-	-	0.29	-	-	0.22	0.22	0.35	0.36	0.42	-	-	0.10	0.10	2.13
*LTL2*	G26422	-	-	-	-	-	-	-	0.11	0.55	0.23	0.36	0.33	0.11	-	0.29	0.19	2.19
*ZEP1*	G45845	0.08	-	-	-	-	-	-	0.22	-	-	0.24	0.58	-	0.28	0.10	-	1.50
*ZEP2*	C962	0.25	0.44	-	-	0.14	-	-	0.11	1.11	0.94	0.12	0.33	0.22	-	0.10	0.10	**3.85**
	S1206																	
	G5928																	
*ZEP3*	G10970S1048	0.25	1.32	0.64	0.62	-	0.53	-	0.22	-	-	0.24	0.83	0.54	-	-	-	**5.19**
*VDE*	G51703	-	-	-	-	-	0.27	-	0.11	0.11	-	0.12	0.33	0.11	-	-	-	1.05
*VDL1*	G36048	0.16	0.22	0.24	0.21	0.14	0.13	0.28	-	0.11	0.12	-	0.17	0.33	0.56	-	0.10	**2.76**
*VDL2*	G45846	-	-	-	-	-	-	-	-	0.22	-	0.12	0.08	-	-	0.39	0.10	0.91
*VDR*	G43240	-	-	-	-	-	-	-	-	0.22	0.12	-	-	0.11	-	-	-	0.45
**total (‰)**		1.48	**2.64**	1.19	1.45	0.57	1.33	0.55	1.10	**2.88**	**2.57**	**2.54**	**3.99**	1.73	1.13	1.07	0.97	
**size library**		12136	4544	12566	4821	6968	7508	3632	9122	9031	8552	8264	12045	9227	3541	10307	10283	

The database was searched for ESTs encoding genes of the diatom carotenoid biosynthetic pathway (see text for abbreviations of the genes) and their distribution in the different libraries was determined. The numbers given in the table reflect the amount of ESTs per one thousand ESTs (‰) for each respective library. Also given are the EST contig ID numbers from the Gene Expression Database v3.0 and the total number of ESTs in each library. Abbreviations used for the libraries: OS) Original 12000 Standard, OM) Oval Morphotype in 10% seawater, TM) Triradiate Morphotype, TA) Tropical Accession at 15C°, SM) Silica Minus in artifical seawater, SP) Silica Plus in artificial seawater with 350 uM metasilicate, NR) Nitrate Replete with 1.12 mM nitrate in chemostat, NS) Nitrate Starved for 3 days with 50 µM nitrate in chemostat, AA) Ammonium Adapted at 75 µM ammonium, UA) Urea Adapted at 50 µM urea, FS) Iron Starved at 5 nM iron, BL) Blue Light for 1 h on 48 h dark adapted cells, LD) Low Decadienal Treated for 6 h with 0.5 ug/ml 2E,4E-decadienal, HD) High Decadienal Treated for 6 h with 5 ug/ml 2E,4E-decadienal, C1) 230 µM/Kg of CO_2_for 1 day in chemostat, C4) 230 µM/Kg of CO_2_ for 4 days in chemostat. Note: this table gives *LTL11* and *LTL2* as alternative genes involved in β-carotene hydroxylation, because *BCH* is absent in *P. tricornutum.*

The blue light (BL) library was found to be the most enriched in carotenogenesis-related ESTs. This library was generated from dark-adapted cells that were treated with 1 h of blue light with an intensity of 25 µmol m^−2^ s^−1^ prior to RNA extraction, and it is enriched in ESTs related to photosynthesis and carbon fixation. The libraries from ammonium adapted (AA), urea adapted (UA) and iron limited (FL) cells are also rich in carotenogenesis-related ESTs compared to the other libraries. The cells of these libraries probably suffered a degree of photosynthetic inhibition due to nutrient shortage [58]–[60]. The ‘carotenogenesis-rich’ libraries all have a relatively high level of LTL1 and LTL2-encoding ESTs. These ESTs may encode Lut-like proteins involved in the hydroxylation of β-carotene (Supplementary Information S1). There are also some substantial differences in the EST distribution within the ‘carotenogenesis-rich’ libraries. For instance, the Fe-limited library does not contain many xanthophyll cycle-related ESTs but instead contains a high number of *PSY*-encoding sequences. The UA and AA libraries are particularly rich in *ZEP2*-encoding ESTs but do not contain any transcripts of the other two *ZEPs*, and the opposite is true for the BL library, which contains a high number of *ZEP1* and *ZEP3* but relatively few *ZEP2* transcripts (Table 2). The BL library is also relatively rich in *VDE* ESTs. Interestingly, cells grown in the AA and UA conditions contain significantly more diadinoxanthin than cells grown under standard nitrogen conditions (Andrew E. Allen, personal communication), whereas blue light treated cells are relatively enriched in violaxanthin (unpublished data).

The finding that the *P. tricornutum* blue light library was enriched in carotenoid biosynthesis-related ESTs as compared to all other libraries suggested that light may play an important role in controlling diatom carotenoid biosynthesis. Whereas the regulatory role of light in the nuclear gene expression of plastid-targeted proteins has been studied for decades in green algae and higher plants, study of these processes in diatoms is still in its infancy. To further explore the regulatory role of light in diatoms at the transcriptional level, we studied the gene expression profiles of *PSY*, *PDS1* and the different xanthophyll cycle genes in response to different light signals. To compare, we also studied the transcript levels of two diatom chlorophyll/carotenoid-binding LHC proteins, fucoxanthin/chlorophyll *a/c*-binding protein B (FCPB) and a protein denoted as ELIP-like, which shares structural similarity to early light-inducible proteins (ELIPs). Like the carotenoid biosynthesis-related genes, the transcripts of these nuclear genes are targeted to the plastid, and in higher plants and green algae the transcription of these genes are known to be regulated at the transcriptional level by light [61], [62]. Likewise, the transcription of FCPs are also known to be light-regulated in diatoms [9], [63].

**Figure 6:**
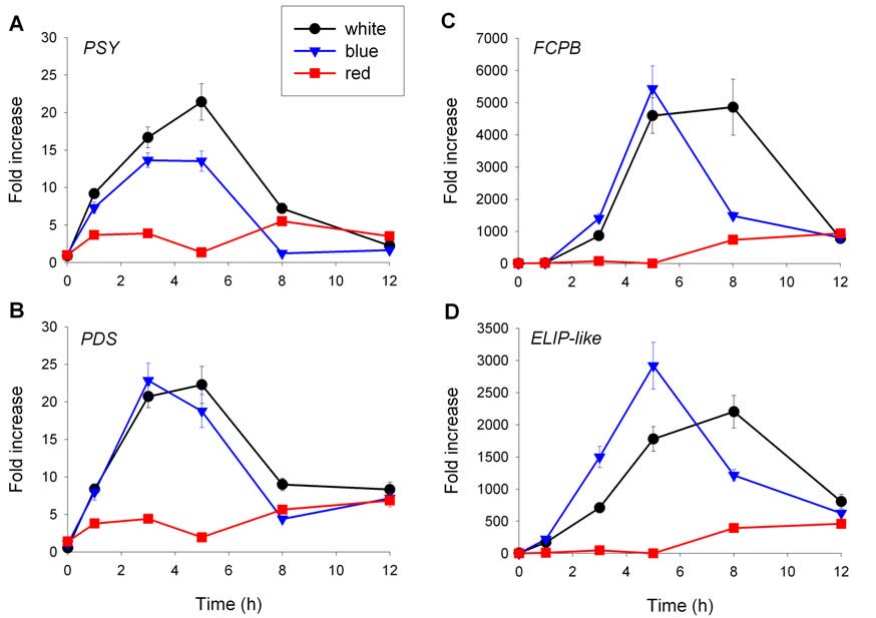
mRNA levels of carotenoid biosynthesis and LHC-related genes upon white, blue or red light stimulation. 48-hour-dark-adapted *P. tricornutum* cells were exposed to 175 µmol m^−2^ s^−1^ continuous white light, or 25 µmol m^−2^ s^−1^ continuous blue or red light and the relative transcript levels of *PSY* (A), *PDS1* (B), *FCPB* (C) and *ELIP-like* (D) were determined after 1, 3, 5, 8 and 12h by qRT-PCR using *H4* as a reference gene. The values were normalized to the transcript levels in the dark. Data are averages of triplicate measurements. The error bars represent standard deviation.

The steady state transcript levels of 48 hour dark-adapted *P. tricornutum* cells treated with either continuous white (175 µmol m^−2^ s^−1^), blue (25 µmol m^−2^ s^−1^) or red (25 µmol m^−2^ s^−1^) light were determined for 12 subsequent hours by quantitative real-time PCR (qRT-PCR) using *histone H4* (*H4*) as a reference gene [9]. The transcript levels of both *PSY* and *PDS1* increased immediately upon light exposure (Fig. 6), and the highest steady state transcript levels were measured after 3 to 5 h light. Transcript levels decreased again after longer exposure. The transcript levels of *PDS1* and, to a lesser degree, of *PSY* are similar at 25 µmol m^−2^ s^−1^ blue light and 175 µmol m^−2^ s^−1^ white light, indicating that the spectral quality of light plays a major role in the regulation of expression of these genes. This observation is further supported by the fact that red light, with an equal fluence rate as blue light, triggered a much weaker response. The induction of the two genes encoding LHC proteins, *FCPB* and *ELIP-like,* began slightly later than the carotenogenesis-related genes (Fig. 6). However, the amplitude of induction was more than 100-fold higher than for *PSY* and *PDS1*. The spectral quality of light was also of major influence for these genes: the transcript levels of both genes in blue and white light were similar, but the kinetics of increase were faster in blue light than in white light. By contrast, the amplitude and kinetics of transcription in response to red light were much lower.

**Figure 7:**
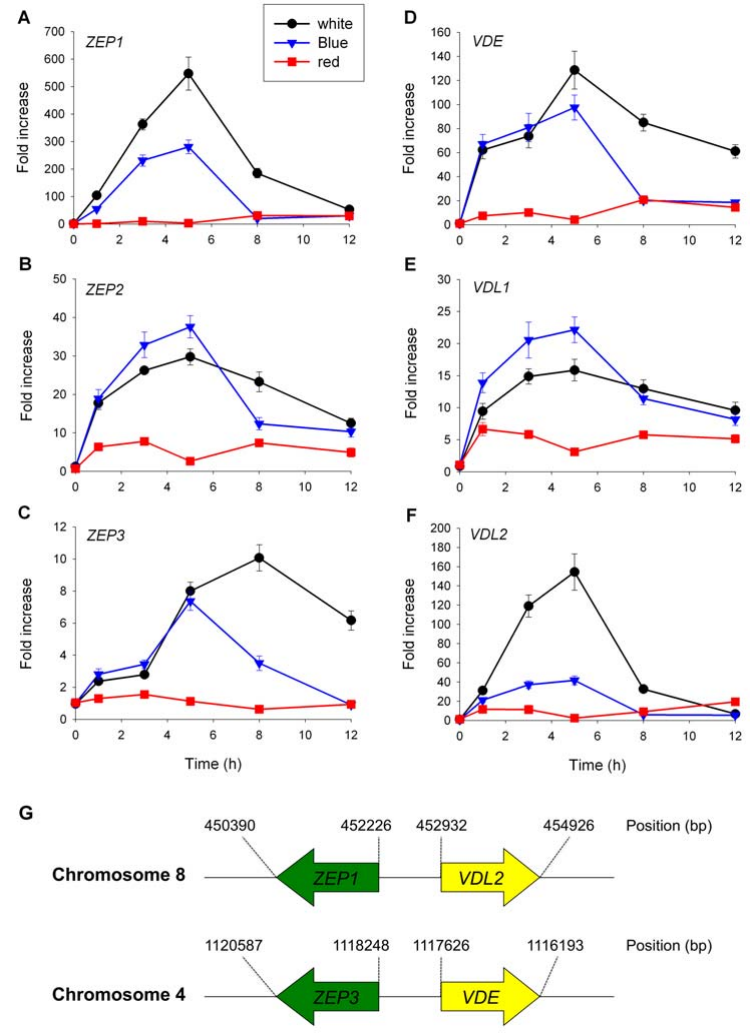
mRNA levels of xanthophyll cycle-related genes upon white, blue or red light stimulation. 48-hour-dark-adapted *P. tricornutum* cells were exposed to 175 µmol m^−2^ s^−1^ continuous white light, or 25 µmol m^−2^ s^−1^ continuous blue or red light and the relative transcript levels of *ZEP1* (A), *ZEP2* (B), *ZEP3* (C), *VDE* (D), *VDL1* (E) and *VDL2* (F) were determined after 1, 3, 5, 8 and 12h by qPCR using *H4* as a reference gene. The values were normalized to the transcript levels in the dark. Data are averages of triplicate measurements. The error bars represent standard deviation.

We also determined the steady state transcript levels of the *P. tricornutum ZEPs* and *VDEs* in 48 h dark-adapted cells subsequently shifted to either white, blue and red light as described above (Fig. 7). We found a steady and strong increase of *ZEP1* transcript levels after 5 hours of white and blue light, and after this period the levels decreased again. The effect of white light was stronger than blue light, and red light did not result in a significant induction (Fig. 7A). The increase in *ZEP2* levels is approximately 10-fold lower than *ZEP1*, but the kinetics of *ZEP2* accumulation is faster and maximal levels were reached within 5 h of illumination (Fig. 7B). Blue light appears to have a stronger effect on *ZEP2* transcription than white light, even though the white light fluence rate was 7 times higher. Contrary to *ZEP1*, red light also has a slight effect on *ZEP2* transcript levels. *ZEP3* mRNA accumulation showed different kinetics, in which a minor induction after 1 h white or blue light was followed by a 2 h lag-phase (Fig. 7C). Maximal *ZEP3* transcript levels were found after 5 h of blue light and after 8 h of white light. *VDE* transcript levels very rapidly increased after stimulation with blue and white light and close to maximal levels were reached within 1h (Fig. 7D). The overall kinetics of steady-state *VDE* mRNA levels was much like *ZEP3* (Fig. 7C,D). These two genes are located next to each other on chromosome 8 (Fig. 7G) and are likely to form a co-regulated gene-cluster. Another such gene cluster is found on chromosome 4 composed of *ZEP1* and *VDL2*, and also in this case the transcript levels of the two clustered genes were comparable (Fig. 7A,F,G).

The data presented shows that mRNA levels for the first two genes committed to carotenoid biosynthesis, as well as the xanthophyll cycle-related genes, rapidly increase upon light exposure and that light of different spectral quality has differential effects. In higher plants and *C. reinhardtii, PSY* and *PDS* transcription has been demonstrated to be regulated through photoreceptors [64]–[66], and the genomes of *T. pseudonana* and *P. tricornutum* contain several putative photoreceptor-encoding genes, including a phytochrome, an animal-type cryptochrome/photolyase and several cryptochrome-DASH homologs [6], [8], [67], which may be important for diatom light-dependent gene-regulation. This is supported, at least for the *PSY* gene, by the finding that a short pulse (5 min) of blue light was sufficient to effectively induce its expression (Fig. S2).

### Conclusions

In conclusion, we propose that both genes encoding enzymes of the xanthophyll cycle (VDE and ZEP) are eukaryotic innovations even though they are putatively located in the primary plastids of plants and the secondary plastids of diatoms. Genes for VDE and VDR have arisen through gene duplication events preceding secondary endosymbiosis. VDL proteins appeared due to a second duplication of VDR, which probably happened exclusively in organisms derived from secondary endosymbiosis. The ZEP genes are also duplicated and again this duplication likely occurred before the secondary endosymbiotic event. A single gene for ZEP from the ciliate *T. thermophila* has also been found, providing the first evidence for a secondary plastid gene being encoded in a heterotrophic ciliate.

The transcription of all *P. tricornutum* xanthophyll cycle-related genes was up-regulated by blue and white light. However, the amplitude and kinetics of mRNA accumulation varied between the different genes and it is possible that their transcription is regulated at multiple levels including photoreceptor- and plastid retrograde signaling mechanisms. The differential expression of *ZEP* and *VDE/VDL* homologs may be indicative of distinct roles for each gene product. Both *ZEP1/VDL2* and *ZEP3/VDE* form gene clusters and appear to be co-regulated upon light stimulation. These genes are not clustered in the *T. pseudonana* genome, providing an illustration of the continued divergence of this gene family even within the diatoms.

## Materials and Methods

### Identification, phylogeny and EST-distribution of carotenogenesis-related genes

The *P. tricornutum* genome sequence, available at the DOE Joint Genome Institute website (http://genome.jgi-psf.org/Phatr2), was analyzed for the presence of genes involved in the carotenoid biosynthetic pathway using BLAST- and keyword searches. Using known sequences from other organisms and *P. tricornutum* EST sequence data available in the diatom EST database (http://www.biologie.ens.fr/diatomics/EST3), the best gene models were selected and annotated. The encoded genes were scanned for the presence of a signal peptide by SignalP v3.0 (http://www.cbs.dtu.dk/services/SignalP) and a plastid transit peptide (cTP) with ChloroP v1.1 (http://www.cbs.dtu.dk/services/ChloroP) [34]. The *P. tricornutum* sequences were used to identify homologs in the *T. pseudonana* genome (http://genome.jgi-psf.org//Thaps3), and keyword- and BLAST searches with sequences from other organisms were also performed. The identity and similarity of the *P. tricornutum* and *T. pseudonana* sequences were determined after ClustalW alignment of the mature protein sequences using Bioedit v7.0.5.3 and the BLOSUM62 similarity matrix [68]. The identity/similarity of the diatom sequences with the best BLASTP hits on the non-redundant protein sequences (nr) deposited at the National Center for Biotechnology Information (http://www.ncbi.nlm.nih.gov/) were also determined.

For phylogenetic analysis, amino acid sequences were aligned using Kalign [69] at the EBI Tools Website (http://www.ebi.ac.uk/kalign/). Gaps and ambiguously aligned regions were excluded from further analysis. Phylogenetic trees were constructed using Maximum likelihood (ML) and Neighbor-joining (NJ) methods as implemented in PhyML [70] and AsaturA [40], respectively. ML trees were computed with particular models of amino acid substitutions chosen according to PROTTEST AIC results [70], [71]. ML bootstraps were inferred from 300 replicates using the same parameters. NJ trees were constructed using the same substitutional model as for ML computations, with NJ bootstraps calculated from 1000 replicates. The majority of the sequences used for phylogenetic analysis were derived from the NCBI database, but the genomes of *Ostreococcus tauri* and *O. lucimarinus* are available at JGI, the genome browser of *Cyanidioschyzon merolae* (http://merolae.biol.s.u-tokyo.ac.jp), the Taxonomically Broad EST Database (http://tbestdb.bcm.umontreal.ca), and the sequence database of the J. Craig Venter Institute (http://www.jcvi.org/gme) were also searched for homologous genes.

The *Phaeodactylum* Digital Gene Expression Database (v3.0) (http://www.biologie.ens.fr/diatomics/EST3) was used to gain insight into the differential expression of carotenogenesis-related genes. The BLASTN search algorithm linked to the database was used to identify the consensus sequence (contig) of multiple ESTs encoding the gene of interest, and the frequency and distribution of the ESTs within each contig was determined. When genes were represented by more than one contig, the accuracy of the contigs was verified using the genome sequence and all gene-specific ESTs were taken into account.

### Strains and growth conditions

*P. tricornutum* Bohlin clone Pt1 8.6 (CCMP 2561) was obtained from the culture collection of the Provasoli-Guillard National Center for Culture of Marine Phytoplankton, Bigelow Laboratory for Ocean Sciences, USA. Cells were grown at 18°C under white fluorescent lights (TLD 58W/840, Philips) at approximately 175 µmol m^−2^ s^−1^ in a 12 hr photoperiod, using f/2 medium made with 0.2-µm-filtered and autoclaved local seawater supplemented with inorganic nutrients and vitamins according to Guillard [72]. Sterility was monitored microscopically and by occasional inoculation into peptone-enriched media to check for bacterial growth in darkness. To study the effect of light quality on steady state gene transcription, 3 L cultures were grown up to a cell density of 2.10^6^ cells/mL as determined by microscopy using a Malassez counting chamber (Brand). The cultures were dark-adapted for 48 hours and subsequently submitted to either blue light (25 µmol m^−2^ s^−1^) with an emission between 380–450 nm (F 40 BB, Philips), red light (25 µmol m^−2^ s^−1^) provided by an LED lamp with a peak emission at 670 nm (QB1310CS-670-735, Quantum devices Inc.), or white fluorescent light (175 µmol m^−2^ s^−1^; TLD 58W/840, Philips). Samples for RNA extraction were collected at the end of the dark period and after the onset of light by centrifugation and the cell pellets were washed with PBS (1x) prior to storage in liquid nitrogen.

### Quantitative real-time RT-PCR

Total RNA was isolated from 1.10^8^ cells using 1.5 mL TriPure isolation reagent (Roche) according to the instructions of the manufacturer. The RNA concentrations were determined photometrically at 260 nm and the integrity was confirmed by agarose gel electrophoresis. Residual genomic DNA was removed by incubating 1 µg of RNA with 1 U/µL amplification grade DNaseI (Invitrogen) at 25°C for 10 min. First-strand cDNA synthesis for quantitative real-time RT-PCR (qRT-PCR) was obtained by incubating 430 ng of DNase1-treated total RNA with 50 ng of random hexamers, 500 µM dNTPs, and 50 U of SuperScript II reverse transcriptase (Invitrogen) in a 30 µL reaction volume (1x RT buffer; Invitrogen) at 25°C for 10 min, followed by a 50 min incubation at 42°C. The reaction was stopped by heat inactivation at 70°C for 15 min, and RNA was removed with an RNase H treatment at 37°C for 20 min.

Gene-specific primers were designed with Primer3 (http://frodo.wi.mit.edu) and are listed in Supplementary Table S1. All primer pairs were initially tested by standard RT-PCR and the amplification of single products with the correct size was verified on 2% (w/v) agarose gels. Real Time-RT PCR amplification mixtures (25 µL) contained 1 µL of cDNA obtained after the reverse transcription (estimated to represent 10 ng), 200 nM forward and reverse primers, and 2x FastStart SYBR Green I PCR Master Mix (Roche). Triplicate reactions were run in an Opticon Chromo4 MJ Research Thermal Cycler (Bio-Rad) in Low-Profile 0.2 mL PCR 8-Tube white Strips (Bio-Rad). The cycling conditions comprised 10 min polymerase activation at 95°C and 40 cycles at 95°C for 15 sec and 60°C for 60 sec. The reaction was ended with a 5 min final elongation at 72°C. Amplicon dissociation curves, i.e., melting curves, were recorded after cycle 40 by heating from 60 to 95°C with a ramp speed of 0.5°C every second which served to confirm primer specificity. The results obtained in the Chromo4 Sequence Detector were exported as tab delimited text files and imported into Microsoft Excel for further analysis. Primer efficiencies were verified by real time RT-PCR on serial dilutions of cDNA. The relative steady state mRNA transcript levels were calculated by comparing the cycle threshold (C_T_) values of the target and the *histone H4*-control transcripts throughout the time-course (time x) with the C_T_ values of these transcripts at 48 hours dark (time 0) using the 2^−**ΔΔ**CT^ method [73]. **ΔΔ**C_T_ is represented by the following formula: ***ΔΔ*** *C_T_ = (C_T,target_−C_T,control__gene_) _time x_−(C_T,target_−C_T,control__gene_) _time 0_*

## Supporting Information

Supplementary Information S1 Formation of Î^2^-carotene and zeaxanthin
(0.07 MB DOC)

Table S1 Primers used for quantitative real-time RT-PCR
(0.06 MB DOC)

Figure S1 ClustalW amino acid sequence alignment of diatom LTLs with LUT proteins from A. thaliana Amino acids shaded in black are identical, and those shaded in grey are similar (BLOSUM62). Sequence accession numbers (NCBI) are as follows: A. thaliana (At) Lut1, NP_190881; Lut5, NP_564384 and Skeletonema costatum (Sc) P450, AAL73435. Protein ID numbers of P. tricornutum (Pt; Phatr v2.0) are: LTL1, 50101 and LTL2, 26422 and protein ID numbers of T. pseudonana (Tp; Thaps v3.0) are: LTL1, 9541 and LTL2, 36235.
(2.60 MB TIF)

Figure S2 PSY and PDS1 gene transcription following a 5 minute blue light pulse 60-hour-dark-adapted P. tricornutum cells were exposed for 5 min to 25 µmol m-2 s-1 blue light and subsequently transferred back to darkness. The relative transcript levels of PSY and PDS1 were determined after 30 min and 1, 3, 5 and 7h by qRT-PCR using H4 as a reference gene. The values were normalized to the transcript levels in the dark. Data are averages of triplicate measurements. The error bars represent standard deviation.
(0.09 MB TIF)
