# Supplementary material for: Evolutionary Origins and Functions of the Carotenoid Biosynthetic Pathway in Marine Diatoms
Source: PLoS One. 2008 Aug 6;3(8):e2896. doi: 10.1371/journal.pone.0002896 (PMC2483416; doi:10.1371/journal.pone.0002896)
Supplement: Supplementary Information S1 — Formation of Î2-carotene and zeaxanthin (0.07 MB DOC) [file pone.0002896.s001.doc]

**Supplementary Information**

**Formation of β-carotene**

The enzyme phytoene synthase (PSY) is responsible for the first committed step of the carotenoid biosynthetic pathway (Fig. 1) and is conserved between archaea, bacteria and eukaryotes [1]. We found one candidate PSY-encoding gene in the *P. tricornutum* genome and two candidate genes in the *T. pseudonana* genome. The diatom PSY1 amino acid sequence, shared by both *P. tricornutum* and *T. pseudonana*, is more similar to PSY sequences from red and brown algae than to homologs in green algae and plants (Table 1 and data not shown).

Phytoene is converted to lycopene in four subsequent desaturation reactions (Fig. 1). In non-photosynthetic bacteria these reactions are all catalyzed by one desaturase protein, named CrtI. In cyanobacteria and photosynthetic eukaryotes two enzymes are involved in lycopene production. The first two desaturation reactions are catalyzed by phytoene desaturase (PDS; CrtP in cyanobacteria) and leads to the formation of ζ-carotene. The last two desaturations are performed by ζ-carotene desaturase (ZDS; CrtQ in cyanobacteria) and lead to the formation of lycopene in the poly-*cis* configuration, called pro-lycopene. The *crtP* and *crtQ* genes were newly acquired in cyanobacteria and subsequently maintained in photosynthetic eukaryotes, and phylogenetic analysis showed that these proteins may have evolved originally from bacterial desaturases involved in the formation of aromatic end groups [1]. Both the *P. tricornutum* and *T. pseudonana* genomes contain two PDS-encoding genes and one ZDS-encoding gene related to the cyanobacterial/plant desaturases (Table 1 and data not shown). A set of 3 genes belonging to the amine oxidase family, which share homology with cyanobacterial ξ-carotene desaturase-related sequences, were also identified but because we do not know whether these genes are relevant for carotenoid biosynthesis they were not further pursued.

Carotenoid isomerases (CRTISO) of cyanobacteria and plants are phylogenetically related to the bacterial CrtI proteins [1]. These proteins are involved in the isomerization of pro-lycopene to the all-*trans* configuration of lycopene, which is necessary in Cyanobacteria and plants because the steric arrangement of pro-lycopene cannot be cyclised by lycopene cyclase. Both diatom genomes contain six putative genes with similarity to *CRTISO,* as well as a related gene model which is more similar to the bacterial *crtI* gene (data not shown).

The enzyme lycopene β-cyclase (LCYB), also known as CrtL-B in Cyanobacteria, can introduce *β*-ionon rings at both ends of the linear lycopene molecule which lead to the formation of β-carotene (β,β-carotene; Fig. 1). In a similar reaction, the enzyme lycopene ε-cyclase (LCYE), also known as CrtL-E in cyanobacteria, can introduce a single ε-ionon ring and together with LCYB it is responsible for the formation of α-carotene (β,ε-carotene). Whereas β-carotene and its derivative zeaxanthin can be found in virtually all photosynthetic organisms, the distribution of α-carotene and its derivative lutein is less ubiquitous. LCYB and LCYE are very similar at the amino acid level, and LCYE probably arose by a gene duplication of LCYB [1]. As Cunningham *et al.* [2] pointed out, such a gene-duplication event appears to be ‘fossilized’ in the genomes of two algae belonging to the prasinophyte genus *Ostreococcus*, because the putative LCYB and LCYE-encoding genes are located in tandem. The same authors found the *LCYE* gene to be absent in the genome of the rhodophyte *Cyanidioschyzon merolae*, which was not surprising since this particular alga does not produce α-carotene and its derivatives. We identified one gene with similarity to *LCYB* in both the *P. tricornutum* and *T. pseudonana* genomes (Table 1). A putative LCYE-encoding gene was not found and its absence reflects the notion that diatoms, like *C. merolae*, do not produce carotenoids with ε-ionon rings. Very recently, a new family of lycopene cyclases was identified in photosynthetic bacteria [3]. This family consists of two types of genes, designated as *cruA* and *cruP*, which are members of the FixC dehydrogenase superfamily. *CruA* and *cruP* are distantly related to *LCY*/*crtL* and can be found in green sulfur bacteria and in cyanobacteria lacking *crtL*. A *cruP* ortholog is also found in the genomes of higher plants, but the gene product still needs biochemical characterization [3]. The diatom genomes also contain a copy of the *cruP* gene (protein ID Phatr 2.0: 3296 and Thaps 3.0: 22130), but they were not further pursued in this study.

**Formation of zeaxanthin**

Zeaxanthin is synthesized from β-carotene by a non-heme di-iron β-ring mono-oxygenase, called β-carotene hydroxylase (BCH), which adds a hydroxyl group to both the β-rings [Fig. 1; 4,5]. In cyanobacteria this reaction is performed by a CrtR-type hydroxylase which shares sequence homology with bacterial β-carotene ketolase proteins, whereas in plants and green algae this enzyme is unrelated to CrtR and instead shares sequence homology with β-carotene hydroxylases of zeaxanthin-producing non-photosynthetic bacteria [6]. Therefore, the evolutionary history of BCH in plants and green algae is not yet well understood.

A 421 bp sequence with homology to the plant and bacterial *BCH* has been identified in the *T. pseudonana* genome [7]. The BCH-encoding gene model is missing the plant-like N-terminal region (± 150 amino acids) and there is no stop codon at the predicted site. The nearest in-frame stop codon was found 1100 bp downstream of the gene model and transcription of this sequence would lead to a long C-terminal tail containing 9 transmembrane regions. This unusual C-terminal region does not show any similarity with other sequences in NCBI. In order to improve the *TpBCH* gene model, the diatom EST database ([http://www.biologie.ens.fr/ diatomics/EST](http://www.biologie.ens.fr/diatomics/EST)) was searched, but no positive hits were found. Extended searches in EST collections from other chlorophyll *c*-containing algae deposited at NCBI were also not fruitful, and we were unable to identify a BCH-encoding gene in the *P. tricornutum* genome. It therefore appears that the *T. pseudonana* BCH gene model encodes either a pseudogene or a protein with an unrelated function. Curiously, *BCH* was also found to be absent in the nuclear genome of *C. merolae*, but Cunningham *et al.* [2] identified a putative cyanobacterial-like β-carotene hydroxylase (CrtR) encoding sequence in the chloroplast genomes of three primitive red algae *C. merolae*, *Galdiera sulphuraria*, and *Cyanidium caldarium*. This gene, however, was found to be absent in the chloroplast genomes of three other red algal species (*Porphyra yezoensis*, *P. purpurea*, and *Gracilaria tenuistipitata*) and the functionality of the *C. merolae* *crtR* gene has still not been demonstrated, despite extensive efforts [2]. Searches for the *crtR* gene in the two diatom nuclear and plastid genomes showed that diatoms, like green algae and plants, have completely lost the CrtR-encoding sequence and, taken together, this data may indicate that both the red and the brown algal lineages have evolved different ways to synthesize zeaxanthin.

The hydroxylation of α-carotene (β,ε-carotene) to lutein has been recently elucidated using *A. thaliana* lutein-deficient mutants [8,9]. Lutein is the most abundant carotenoid in green photosynthetic organisms and it plays an important role in the proper folding of Lhc proteins and in the quenching of chlorophyll triplets in the LHCII complex [10]. The proteins involved in the hydroxylation of the β-ring (LUT5, also called CYP97A3) and the ε-ring (LUT1, also called CYP97C1) of α-carotene were shown to be structurally unrelated to BCH and instead were identified as heme-containing monooxygenases belonging to the cytochrome P450 protein family. Both proteins also showed a weak ability to hydroxylate the β-rings of β-carotene [6,8,9]. Even though diatoms do not produce α-carotene and lutein [11], two genes with similarity to *LUT* were found in the genomes of both *T. pseudonana* and *P. tricornutum* (Fig. S1). The diatom LUTlike-encoding genes (*LTL*) all contain a putative signal peptide and may be targeted to the plastid (Table 1). According to the available EST data, the N-terminal sequence of PtLTL2 is almost 90 amino acids longer than the other LUT sequences and the TpLTL2 gene-model appears to be incomplete. Although still to be experimentally proven, it may be possible that β-carotene hydroxylation in diatoms is catalyzed by plastid-localized β-carotene-specialized P450 proteins. This assumption seems reasonable since a P450-type protein has already been implicated in the hydroxylation of β-carotene in the bacterium *Thermus thermophilus* [12]. The LTL-encoding genes may be good candidates to catalyze this reaction in diatoms because of their homology to plant LUT proteins (Fig. S1). Because P450 proteins are unrelated to the non-heme di-iron monooxygenase BCH and CrtR, the putative use of these proteins in *P. tricornutum* and *T. pseudonana* would imply that biosynthesis of zeaxanthin in diatoms has evolved independently from plants and also independently from cyanobacteria. The replacement of a di-iron-monooxygenase with a heme-containing monooxygenase (one recyclable Fe-ion per heme molecule) may be of substantial benefit for diatoms, because it has now been well established that the bioavailability of iron in the ocean is scarce and that diatoms are particularly sensitive to iron concentrations [13-17].

**References**

1. Sandmann G (2002) Molecular evolution of carotenoid biosynthesis from bacteria to plants. Physiologia Plantarum 116: 431-440.

2. Cunningham FX, Jr., Lee H, Gantt E (2007) Carotenoid biosynthesis in the primitive red alga *Cyanidioschyzon merolae*. Eukaryot Cell 6: 533-545.

3. Maresca JA, Graham JE, Wu M, Eisen JA, Bryant DA (2007) Identification of a fourth family of lycopene cyclases in photosynthetic bacteria. Proc Natl Acad Sci U S A 104: 11784-11789.

4. Bouvier F, Keller Y, d'Harlingue A, Camara B (1998) Xanthophyll biosynthesis: molecular and functional characterization of carotenoid hydroxylases from pepper fruits (*Capsicum annuum L.*). Biochim Biophys Acta 1391: 320-328.

5. Sun Z, Gantt E, Cunningham FX, Jr. (1996) Cloning and functional analysis of the beta-carotene hydroxylase of *Arabidopsis thaliana*. J Biol Chem 271: 24349-24352.

6. Tian L, DellaPenna D (2004) Progress in understanding the origin and functions of carotenoid hydroxylases in plants. Arch Biochem Biophys 430: 22-29.

7. Wilhelm C, Buchel C, Fisahn J, Goss R, Jakob T, et al. (2006) The regulation of carbon and nutrient assimilation in diatoms is significantly different from green algae. Protist 157: 91-124.

8. Kim J, DellaPenna D (2006) Defining the primary route for lutein synthesis in plants: the role of *Arabidopsis* carotenoid beta-ring hydroxylase CYP97A3. Proc Natl Acad Sci U S A 103: 3474-3479.

9. Tian L, Musetti V, Kim J, Magallanes-Lundback M, DellaPenna D (2004) The *Arabidopsis* LUT1 locus encodes a member of the cytochrome p450 family that is required for carotenoid epsilon-ring hydroxylation activity. Proc Natl Acad Sci U S A 101: 402-407.

10. Dall'Osto L, Lico C, Alric J, Giuliano G, Havaux M, et al. (2006) Lutein is needed for efficient chlorophyll triplet quenching in the major LHCII antenna complex of higher plants and effective photoprotection in vivo under strong light. BMC Plant Biol 6: 32.

11. Strain HH, Manning WM, Hardin G (1944) Xanthophylls and carotenes of diatoms, brown algae, dinoflagellates, and sea-anemones Biol Bull: 169-191.

12. Blasco F, Kauffmann I, Schmid RD (2004) CYP175A1 from *Thermus thermophilus* HB27, the first beta-carotene hydroxylase of the P450 superfamily. Appl Microbiol Biotechnol 64: 671-674.

13. Behrenfeld MJ, Worthington K, Sherrell RM, Chavez FP, Strutton P, et al. (2006) Controls on tropical Pacific Ocean productivity revealed through nutrient stress diagnostics. Nature 442: 1025-1028.

14. Boyd PW, Jickells T, Law CS, Blain S, Boyle EA, et al. (2007) Mesoscale iron enrichment experiments 1993-2005: synthesis and future directions. Science 315: 612-617.

15. Boyd PW, Law CS, Wong CS, Nojiri Y, Tsuda A, et al. (2004) The decline and fate of an iron-induced subarctic phytoplankton bloom. Nature 428: 549-553.

16. Strzepek RF, Harrison PJ (2004) Photosynthetic architecture differs in coastal and oceanic diatoms. Nature 431: 689-692.

17. Tsuda A, Takeda S, Saito H, Nishioka J, Nojiri Y, et al. (2003) A mesoscale iron enrichment in the western subarctic Pacific induces a large centric diatom bloom. Science 300: 958-961.
