# Supplementary material for: Evolutionary Origins and Functions of the Carotenoid Biosynthetic Pathway in Marine Diatoms
Source: PLoS One. 2008 Aug 6;3(8):e2896. doi: 10.1371/journal.pone.0002896 (PMC2483416; doi:10.1371/journal.pone.0002896)
Supplement: Table S1 — Primers used for quantitative real-time RT-PCR (0.06 MB DOC) [file pone.0002896.s002.doc]

Table S1. Primers used for quantitative real-time RT-PCR

| **Gene** | **ID** ^A^ | **Primer** | **Primer sequence (5'-3')** | **Product size (bp)** |
| --- | --- | --- | --- | --- |
| *H4* | 34971 | H4 fw | AGGTCCTTCGCGACAATATC | 151 |
|  |  | H4 rev | ACGGAATCACGAATGACGTT |  |
| *PSY* | 56481 | PSY fw | cacgaagaggtgtattcatgctg | 150 |
|  |  | PSY rev | acagcttttcccacttatccaca |  |
| *PDS1* | 45735 | PDS fw | tctcatgaaccagaaaatgctca | 168 |
|  |  | PDS rev | aagacttcttcgttgatgcgttc |  |
| *ZEP1* | 45845 | ZEP1 fw | CCAGATTCTACTCGGCAAGGAC | 188 |
|  |  | ZEP1 rev | CATTCTGATCTCCTGGCTCCTC |  |
| *ZEP2* | 56488 | ZEP2 fw | TCCGCCGATGTTCTAGTAGGAT | 178 |
|  |  | ZEP2 rev | TTGCATAGTAGTCCGGGGTCTT |  |
| *ZEP3* | 56492 | ZEP3c fw | TCACCACATCCTCAGGGCTA | 186 |
|  |  | ZEP3c rev | CCAATGACAAAAGCATCTTCGAT |  |
| *VDE* | 44635 | VDEb fw | ACAGCATTGGCACTAACGATT | 198 |
|  |  | VDEb rev | TCGTCCCGTACAGGTGTTAATG |  |
| *VDL1* | 46155 | VDL1 fw | TCTACTAGGAGGGACCCCGTTAC | 153 |
|  |  | VDL1 rev | CTCGTTCTTGTTTCACCCATACC |  |
| *VDL2* | 45846 | VDL2 fw | GAATTTTCCATGCCACACTTGTT | 167 |
|  |  | VDL2 rev | GTGGACACTTGGTCGAATACCAT |  |
| *FCPB* | 25172 | FCPB fw | GCCGATATCCCCAATGGATTT | 177 |
|  |  | FCPB rev | CTTGGTCGAAGGAGTCCCATC |  |
| *ELIP-like* | 17326 | ELIP fw | TGTTCGGCTGGTTTTTCATCTG | 258 |
|  |  | ELIP rev | TGGCTTCATAGTTGGGGTGGTT |  |

^A^ Protein IDs from the *P. tricornutum* genome browser v2.0
