# Supplementary figures and images for: Evolutionary Origins and Functions of the Carotenoid Biosynthetic Pathway in Marine Diatoms
Source: PLoS One. 2008 Aug 6;3(8):e2896. doi: 10.1371/journal.pone.0002896 (PMC2483416; doi:10.1371/journal.pone.0002896)

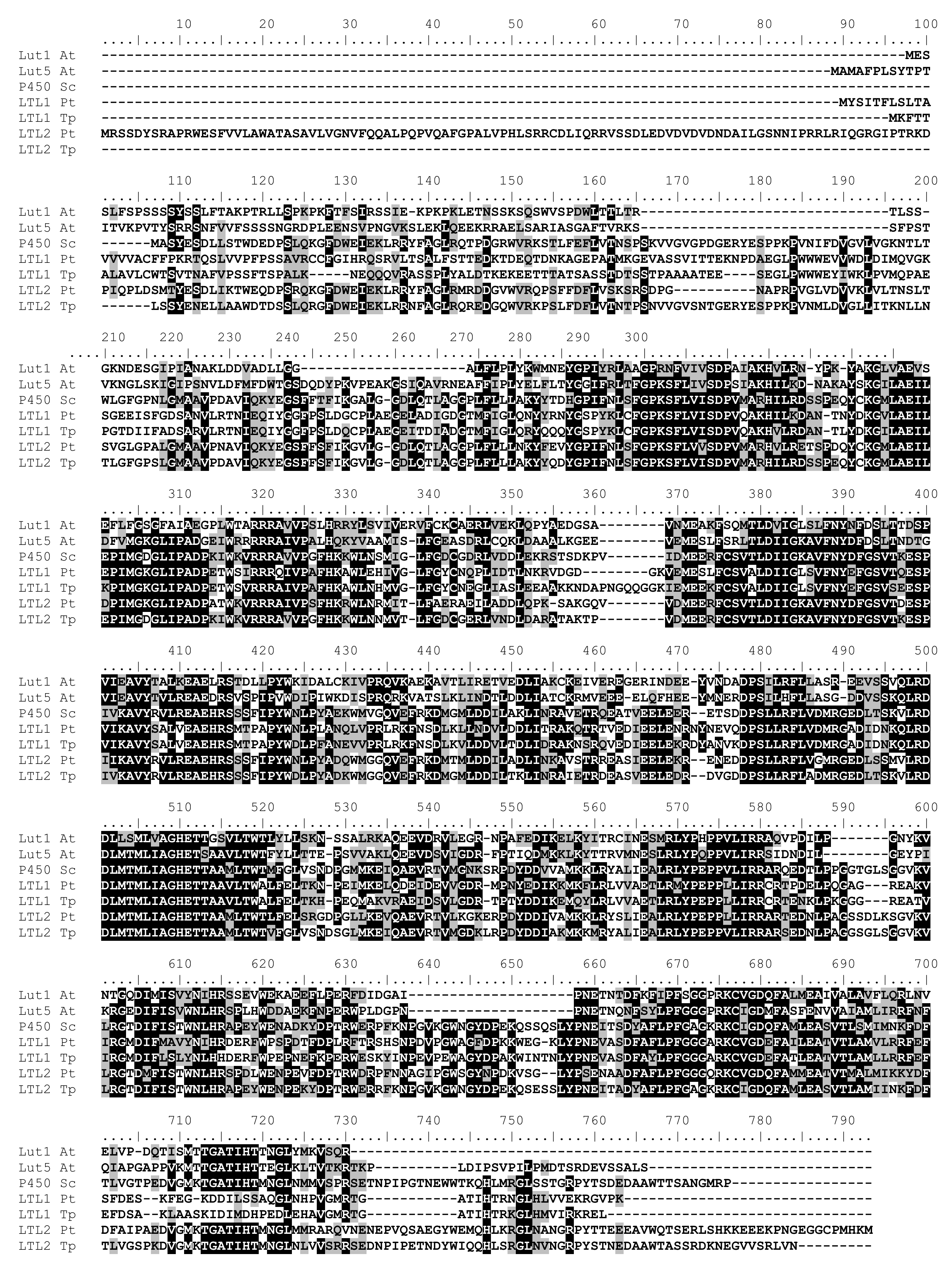

Supplement: Figure S1 — ClustalW amino acid sequence alignment of diatom LTLs with LUT proteins from A. thaliana Amino acids shaded in black are identical, and those shaded in grey are similar (BLOSUM62). Sequence accession numbers (NCBI) are as follows: A. thaliana (At) Lut1, NP_190881; Lut5, NP_564384 and Skeletonema costatum (Sc) P450, AAL73435. Protein ID numbers of P. tricornutum (Pt; Phatr v2.0) are: LTL1, 50101 and LTL2, 26422 and protein ID numbers of T. pseudonana (Tp; Thaps v3.0) are: LTL1, 9541 and LTL2, 36235. (2.60 MB TIF) [file pone.0002896.s003.tif]

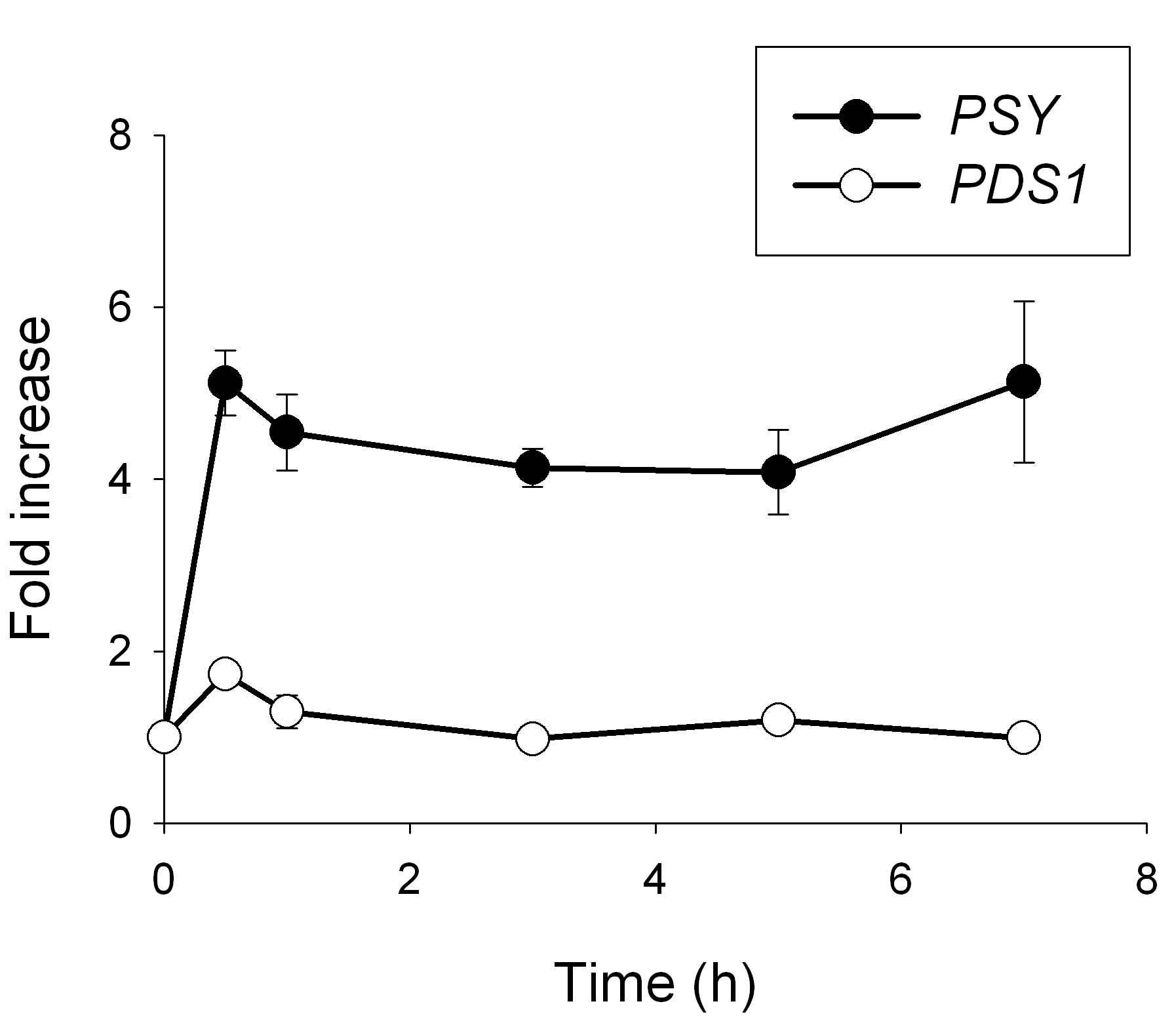

Supplement: Figure S2 — PSY and PDS1 gene transcription following a 5 minute blue light pulse 60-hour-dark-adapted P. tricornutum cells were exposed for 5 min to 25 µmol m-2 s-1 blue light and subsequently transferred back to darkness. The relative transcript levels of PSY and PDS1 were determined after 30 min and 1, 3, 5 and 7h by qRT-PCR using H4 as a reference gene. The values were normalized to the transcript levels in the dark. Data are averages of triplicate measurements. The error bars represent standard deviation. (0.09 MB TIF) [file pone.0002896.s004.tif]
